# Construction of a nomogram model to predict the risk of retinopathy of prematurity reactivate after intravitreal anti-vascular endothelial growth factor therapy: a retrospective study

**DOI:** 10.3389/fped.2024.1440437

**Published:** 2025-01-07

**Authors:** Ziyun Shen, Qingfei Hao, Tiantian Yang, Xiuyong Cheng

**Affiliations:** Department of Neonatology, The First Affiliated Hospital of Zheng Zhou University, Zhengzhou, China

**Keywords:** anti-vascular endothelial growth factor, retinopathy of prematurity, reactivate, nomogram, preterm

## Abstract

**Objective:**

To explore the risk factors for the reactivate of retinopathy of prematurity (ROP) after intravitreal injection of anti-vascular endothelial growth factor (VEGF) and to construct a nomogram model to predict the risk of ROP reactivate.

**Methods:**

A retrospective analysis was conducted on 185 ROP children who underwent anti-VEGF treatment at the First Affiliated Hospital of Zhengzhou University from January 2017 to October 2023. They were randomly divided into a training set (129 cases) and a validation set (56 cases) at a ratio of 7:3. The training set was further divided into a reactivate group (*n* = 18) and a non-reactivate group (*n* = 111) based on whether ROP recurred after treatment. Multivariable logistic regression analysis was used to screen for risk factors for ROP reactivate. A nomogram model was constructed using R software and validated using the validation set. The discrimination, calibration, and clinical net benefit of the model were evaluated using the receiver operating characteristic curve (ROC curve), calibration curve, and decision curve analysis, respectively.

**Results:**

Multivariable logistic regression analysis showed that the number of red blood cell transfusions, use of pulmonary surfactant (PS) 2 times or more, and preoperative fundus hemorrhage were independent risk factors for ROP reactivate (*P* < 0.05). The area under the ROC curve (AUC) of the training set was 0.810 (95% CI: 0.706–0.914), and that of the validation set was 0.756 (95% CI: 0.639–0.873). The Hosmer-Leme show goodness-of-fit test indicated a good fit of the model (*P* = 0.31). Calibration curve analysis and decision curve analysis suggested high predictive efficacy and clinical application value of the model.

**Conclusions:**

The number of red blood cell transfusions, use of PS 2 times or more, and preoperative fundus hemorrhage are independent risk factors for ROP reactivate. The nomogram model constructed based on these factors has high predictive efficacy and clinical application value.

## Introduction

Retinopathy of prematurity (ROP) is a vascular proliferative disease characterized by abnormal development of immature retinal vessels and is a major cause of blindness in children in developing and developed countries (1). With advances in perinatal medicine, the survival rate of premature infants has been continuously increasing, leading to an increasing number of ROP patients (2). Early screening and intervention can effectively improve the prognosis of ROP patients (3, 4). Laser photocoagulation therapy has been the traditional treatment for ROP (5). However, such treatment may lead to permanent retinal damage, with risks of visual field defects, high myopia, and other complications 6). Furthermore, laser photocoagulation may prove to be challenging in cases of poor pupillary dilation and poor visualization secondary to media opacities (7). Anti-vascular endothelial growth factor (VEGF) therapy was first reported for the treatment of ROP in 2007 (8). A multicenter randomized controlled trial in 2011 showed that intravitreal injection of bevacizumab was more effective than laser photocoagulation for stage 3 ROP with plus disease (9). Subsequently, intravitreal injection of anti-VEGF has been widely used in the treatment of ROP (10–12). Compared with laser photocoagulation, anti-VEGF treatment is less invasive, does not require general anesthesia, and does not damage the surrounding retina, thereby reducing the risks of visual field defects and refractive errors. The main advantages of anti-VEGF treatment over conventional laser photocoagulation include promoting rapid regression of acute-phase ROP, allowing potentials for retinal vascularization, approaching eyes with a rigid pupil, and a lower chance of unfavorable outcomes in type 1 ROP in zone I or posterior zone II (13). However, anti-VEGF treatment also carries the risk of ROP reactivate, which may lead to serious consequences such as retinal detachment, visual impairment, and permanent blindness. Therefore, early prediction of the risk of ROP reactivate is helpful for clinicians to develop personalized follow-up and management strategies to improve the prognosis of patients. Currently, there is limited research on the risk of ROP reactivate. This study retrospectively analyzed the clinical data of ROP children treated with intravitreal anti-VEGF therapy, explored the risk factors for ROP reactivate, and constructed a nomogram model to predict the risk of ROP reactivate, providing a basis for early prediction and intervention of ROP reactivate.

## Materials and methods

### Study population

We selected infants diagnosed with retinopathy of prematurity (ROP) who received anti-VEGF therapy for the first time at our hospital from January 2017 to October 2023 as the study population. Inclusion criteria were as follows: (1) Infants diagnosed with ROP according to the international ROP classification criteria (14), (2) The initial treatment modality was an intravitreal injection of anti-VEGF agents. Exclusion criteria were: (1) Incomplete clinical data, (2) Infants with congenital ocular diseases such as retinoblastoma, familial exudative vitreoretinopathy, congenital cataracts, etc. This was a single-center retrospective case series study. Ethical approval was obtained from the First Affiliated Hospital of Zhengzhou University Institutional Review Board (No.: 2023-KY-1374) in accordance with the principles of the Declaration of Helsinki.

### Data collection

We retrospectively collected medical records of enrolled infants, including: (1) General information about the infants: gender, birth weight, gestational age, duration of oxygen therapy, duration of invasive and non-invasive mechanical ventilation, hemoglobin level, number of red blood cell transfusions, number of surfactant (PS) applications, complications of prematurity [respiratory distress syndrome (RDS), sepsis, bronchopulmonary dysplasia (BPD), necrotizing enterocolitis (NEC)], preoperative ROP screening results, anti-VEGF drugs, etc. (2) Maternal antenatal information: method of conception, mode of delivery, multiple gestation, gestational diabetes, gestational hypertension, chorioamnionitis, etc.

### Treatment and follow-up

Intravitreal injection of anti-VEGF drugs: Intravitreal injection of VEGF drugs in all children was performed by the same ophthalmologist. During the study period, the anti-VEGF drugs of choice were ranibizumab, aflibercept, or conbercept. The decision to choose a different anti-VEGF drug was made with an informed consent form completed by the parents and obtained by the ophthalmologist.

Under topical anesthesia, 0.25 mg/0.025 ml of ranitidine, 1 mg/0.025 ml of aflibercept, or 0.25 mg/0.025 ml of conbercept were injected into the vitreal cavity through the folds using a 30-gauge needle inserted 1.0 mm posterior to the limbus of both eyes. After anti-VEGF treatment, qualified ophthalmologists used the wide-angle digital retinal imaging system RetCam III to obtain digital retinal images and record the lesion area, stage, additional lesions, ridge and supracrestal blood vessel regression, etc. Examinations were performed weekly or every 2 weeks, depending on the retinal examination results, and continued until vascularization reached zone III or the identified ROP resolved significantly.

Indications for anti-VEGF therapy include: pre-threshold type 1 ROP, threshold ROP, and rapidly progressive posterior pole ROP. Relevant definitions, diagnosis and classification refer to the international ROP classification standards (14).

ROP reactivation: Following anti-VEGF therapy, new lines or ridges, dilation, or tortuosity of retinal vasculature, or new extraretinal neovascularization is described by the term reactivated at the most anterior ridge (14).

### Grouping of study subjects

Use the sample function to randomly divide the research objects into a training set and a validation set in a ratio of 7:3. The training set was used to analyze the factors influencing ROP reactivate and to construct a nomogram predictive model, while the validation set was used for validation. Based on whether ROP recurred after initial anti-VEGF treatment, both the training and validation sets were further divided into a reactivate group and a non-reactivate group. Reactivate group: Infants who showed regression or disappearance of ridges and reduced additional lesions after initial anti-VEGF treatment but later developed ridge proliferation, neovascularization, or required retreatment during follow-up; Nonreactivate group: Infants who showed reduced additional lesions and disappearance of ridges after initial anti-VEGF treatment, with retinal vessels gradually growing toward the periphery, and complete regression of lesions during follow-up.

### Statistical analysis

Statistical analysis was performed using SPSS version 26.0 software. Normally distributed continuous data were expressed as mean ± standard deviation (SD) and compared using independent samples *t*-test. Non-normally distributed continuous data were expressed as median (interquartile range) [M (P25, P75)] and compared using the Mann-Whitney *U*-test. Categorical data were presented as frequencies and percentages (%) and compared using the chi-square test. Multivariate logistic regression analysis was conducted to identify independent risk factors for ROP reactivate. A nomogram predictive model for ROP reactivate risk was constructed using R software (version 4.2.3). The fit of the model was assessed using the Hosmer-Leme show test. The nomogram model was validated using the validation set. The discriminatory ability of the model was evaluated by the area under the receiver operating characteristic curve (AUC), and the calibration of the model was assessed using calibration curves. Decision curve analysis was performed to evaluate the clinical net benefit of the model. Discrimination and calibration were evaluated using 1,000 repeated bootstrap resampling. All statistical tests were two-sided, and *P* < 0.05 was considered statistically significant.

## Results

### Patient characteristics

From January 2017 to October 2023, a total of 201 infants received intravitreal injections of VEGF therapy for ROP at the First Affiliated Hospital of Zhengzhou University. Six infants had undergone laser treatment at another hospital before coming to our hospital, two infants were diagnosed with congenital glaucoma, and three infants were diagnosed with congenital cataracts. Additionally, five infants were excluded due to incomplete medical records. A total of 185 infants were included in this study, with 129 infants allocated to the training set and 56 infants allocated to the validation set. In the training set, 18 infants experienced ROP reactivate after anti-VEGF treatment. In the training concentration, there were significant differences in gestational age, birth weight, duration of invasive mechanical ventilation, times of red blood cell transfusion, application of ≥2 PS and preoperative fundus hemorrhage between the recurrent group and the non-recurrent group (*P* < 0.05) (Table 1).

**Table 1:** Clinical features in patients.

	Training set (*n* = 129)				Validating set (*n* = 56)			
	No ROP reactivate group (*n* = 111)	ROP reactivate group (*n* = 18)	t/*χ*²	*P*-value	No ROP reactivate group (*n* = 50)	ROP reactivate group (*n* = 6)	t/χ²	*P*-value
GA (week, IQR)	28.72 ± 1.86	27.62 ± 2.51	*t* = 2.22	0.03	28.73 ± 2.01	28.21 ± 1.67	*t* = 0.60	0.55
BW (g, IQR)	1,067.34 ± 274.80	928.89 ± 248.31	*t* = 2.01	0.05	1,097.60 ± 273.05	1,105.00 ± 323.65	*t* = −0.06	0.95
Oxygen inhalation duration (day, IQR)	35.74 ± 25.29	53.06 ± 36.85	*t* = −1.92	0.07	33.33 ± 22.15	41.67 ± 33.40	*t* = −0.82	0.41
Invasive ventilator duration (day, IQR)	11.01 ± 14.37	20.28 ± 23.04	*t* = −2.31	0.02	7.87 ± 11.84	8.50 ± 10.69	*t* = −0.12	0.90
Non invasive ventilator duration (day, IQR)	22.93 ± 12.20	26.56 ± 17.75	*t* = −1.09	0.28	20.18 ± 19.26	24.67 ± 9.87	*t* = −0.56	0.58
Erythrocyte transfusion times (time, IQR)	5.93 ± 4.03	9.89 ± 6.98	*t* = −2.35	0.03	5.22 ± 3.74	6.00 ± 4.86	*t* = −0.47	0.64
Erythrocyte transfusion volume (ml, IQR)	163.35 ± 112.45	263.00 ± 218.99	*t* = −1.89	0.07	143.14 ± 101.87	162.17 ± 116.00	*t* = −0.43	0.67
HGB (g/L, IQR)	106.14 ± 17.50	112.44 ± 17.31	*t* = −1.42	0.16	102.73 ± 17.46	104.75 ± 9.55	*t* = −0.28	0.78
Male, *n* (%)	72 (64.86)	9 (50.00)	χ² = 1.47	0.23	31 (62.00)	2 (33.33)	χ² = 0.83	0.36
Natural conception, *n* (%)	84 (75.68)	12 (66.67)	χ² = 0.27	0.60	45 (90.00)	6 (100.00)	–	1.00
natural labour, *n* (%)	43 (38.74)	7 (38.89)	χ² = 0.00	0.99	15 (30.00)	3 (50.00)	χ² = 0.28	0.60
Multiple pregnancy, *n* (%)	27 (24.32)	3 (16.67)	χ² = 0.17	0.68	13 (26.00)	1 (16.67)	χ² = 0.00	1.00
Gestational hypertension, *n* (%)	46 (41.44)	6 (33.33)	χ² = 0.42	0.52	19 (38.00)	1 (16.67)	χ² = 0.34	0.56
GDM, *n* (%)	17 (15.32)	4 (22.22)	χ² = 0.15	0.70	5 (10.00)	1 (16.67)	–	0.51
Chorioamnionitis,*n* (%)	2 (1.80)	0 (0.00)	–	1.00	3 (6.00)	0 (0.00)	–	1.00
Asphyxia, *n* (%)	58 (52.25)	8 (44.44)	χ² = 0.38	0.54	25 (50.00)	1 (16.67)	χ² = 1.24	0.27
NEC, *n* (%)	16 (14.41)	3 (16.67)	χ² = 0.00	1.00	8 (16.00)	1 (16.67)	–	1.00
RDS, *n* (%)	110 (99.10)	17 (94.44)	–	0.26	48 (96.00)	6 (100.00)	–	1.00
BPD, *n* (%)	78 (70.27)	16 (88.89)	χ² = 1.86	0.17	28 (56.00)	4 (66.67)	χ² = 0.00	0.95
Sepsis, *n* (%)	92 (82.88)	15 (83.33)	χ² = 0.00	1.00	38 (76.00)	4 (66.67)	χ² = 0.00	1.00
Pneumonia, *n* (%)	32 (28.83)	7 (38.89)	χ² = 0.74	0.39	13 (26.00)	1 (16.67)	χ² = 0.00	1.00
Intracranial hemorrhage, *n* (%)	87 (78.38)	16 (88.89)	χ² = 0.51	0.48	42 (84.00)	6 (100.00)	–	0.58
PDA, *n* (%)	44 (39.64)	8 (44.44)	χ² = 0.15	0.70	20 (40.00)	3 (50.00)	χ² = 0.00	0.98
≥2 PS, *n* (%)	27 (24.32)	9 (50.00)	χ² = 5.08	0.02	11 (22.00)	2 (33.33)	χ² = 0.01	0.91
Zone preoperative screening, *n* (%)			χ² = 3.01	0.08			χ² = 0.57	0.45
Ⅰ	38 (34.23)	10 (55.56)			13 (26.00)	3 (50.00)		
Ⅱ	73 (65.77)	8 (44.44)			37 (74.00)	3 (50.00)		
Stage preoperative screening, *n* (%)			–	0.08			–	0.25
1	5 (4.50)	1 (5.56)			4 (8.00)	0 (0.00)		
2	47 (42.34)	5 (27.78)			24 (48.00)	3 (50.00)		
3	58 (52.25)	10 (55.56)			22 (44.00)	2 (33.33)		
4	1 (0.90)	2 (11.11)			0 (0.00)	1 (16.67)		
Plus preoperative screening, *n* (%)	82 (73.87)	16 (88.89)	–	0.42	36 (72.00)	4 (66.67)	–	0.19
Bleed preoperative screening, *n* (%)	8 (7.21)	5 (27.78)	–	0.02	4 (8.00)	2 (33.33)	–	0.12
Anti-VEGF drugs, *n* (%)			χ² = 2.21	0.33			–	0.32
Ranibizumab	43 (38.74)	7 (38.89)			24 (48.00)	1 (16.67)		
Aflibercept	41 (36.94)	4 (22.22)			17 (34.00)	3 (50.00)		
Conbercept	27 (24.32)	7 (38.89)			9 (18.00)	2 (33.33)		

ROP, retinopathy of prematurity; GA, gestational age; BW, birth weight; GDM, gestation diabetes mellitus; NEC,necrotizing enterocolitis; RDS, neonatal respiratory distress syndrome; BPD, bronchopulmonary dysplasia; PDA, patent ductus arteriosus; PS, pulmonary surfactant.

### Establishment of a predictive model

**Figure 1:**
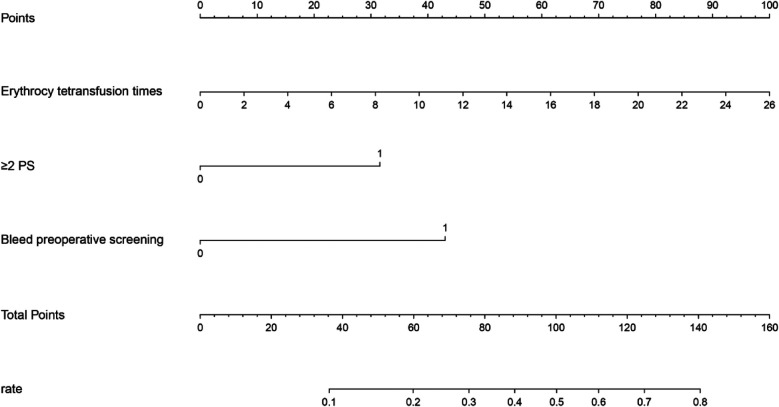
Nomogram for ROP reactivate risk according to the variables, the corresponding scores are obtained, and the risk axis corresponding to the total score is obtained by adding the scores of each variable, so that the risk of ROP reactivate can be obtained.

Single-factor and multi-factor logistic regression analysis showed that the independent risk factors for ROP reactivate were the number of red blood cell transfusions, use of PS 2 or more times, and preoperative fundus hemorrhage (Table 2). Based on the three significant variables identified in the multi-factor logistic regression analysis, a line chart of the risk of ROP reactivate with intravitreal injection of anti-VEGF therapy was plotted using R software (Figure 1).The corresponding scores are obtained according to the variables, and the scores of each variable are added to obtain the total score. The value corresponding to the risk axis of the total score is the probability of ROP reactivation. The higher the total score, the higher the risk of ROP reactivation. For example, a child has 26 red blood cell transfusions (scored 100 points), 2 PS applications (scored 30 points), and no preoperative fundus bleeding (scored 0 points), with a total score of 130 points, which can be predicted based on the nomogram. The probability of ROP reactivation in this child is approximately 74%.

**Table 2:** Univariate and multivariate analysis of factors related to ROP reactivate.

	Univariable models		Multivariable models	
	OR (95%CI)	*P*-value	OR (95%CI)	*P*-value
GA (week)	0.70 (0.51–0.97)	0.03	1.48 (0.86–2.56)	0.16
BW (g)	0.99 (0.99–0.99)	0.05	1.00 (0.99–1.00)	0.14
Invasive ventilator duration (day)	1.03 (1.01–1.06)	0.04	1.01 (0.97–1.05)	0.10
Erythrocyte transfusion times (time)	1.16 (1.05–1.29)	0.00	1.30 (1.05–1.61)	0.02
≥2 PS (*n*)	3.11 (1.12–8.63)	0.03	4.19 (1.07–16.47)	0.04
Bleed preoperative screening (*n*)	5.66 (1.57–20.45)	0.01	14.09 (1.97–100.69)	0.01

GA, gestational age; BW, birth weight; PS, pulmonary surfactant.

### Validation and evaluation of predictive model

**Figure 2:**
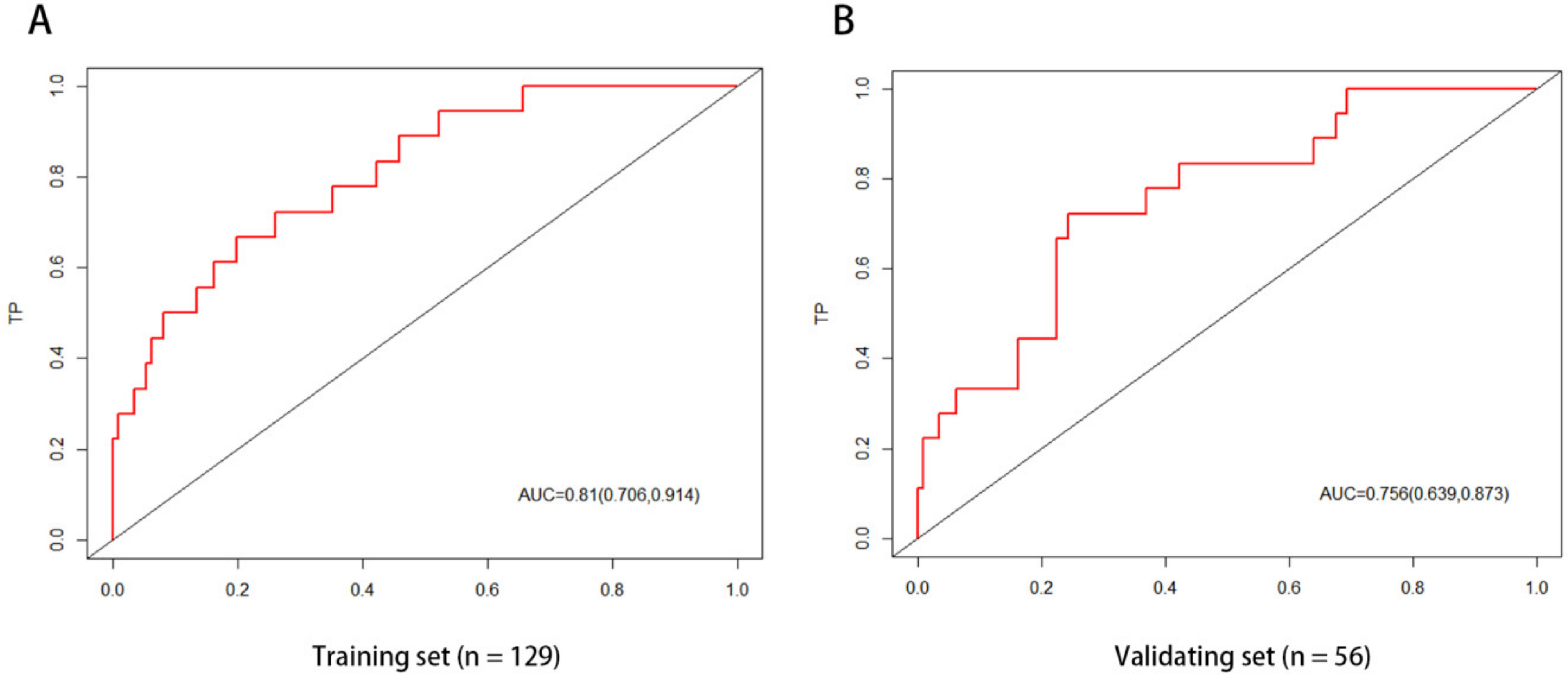
ROC curve of nomogram prediction model the AUCs of the nomogram model in the training set **(A)** and the verification set **(B)** in predicting ROP reactivate is 0.810 and 0.756, respectively, which indicates that the model has good discrimination ability.

**Figure 3:**
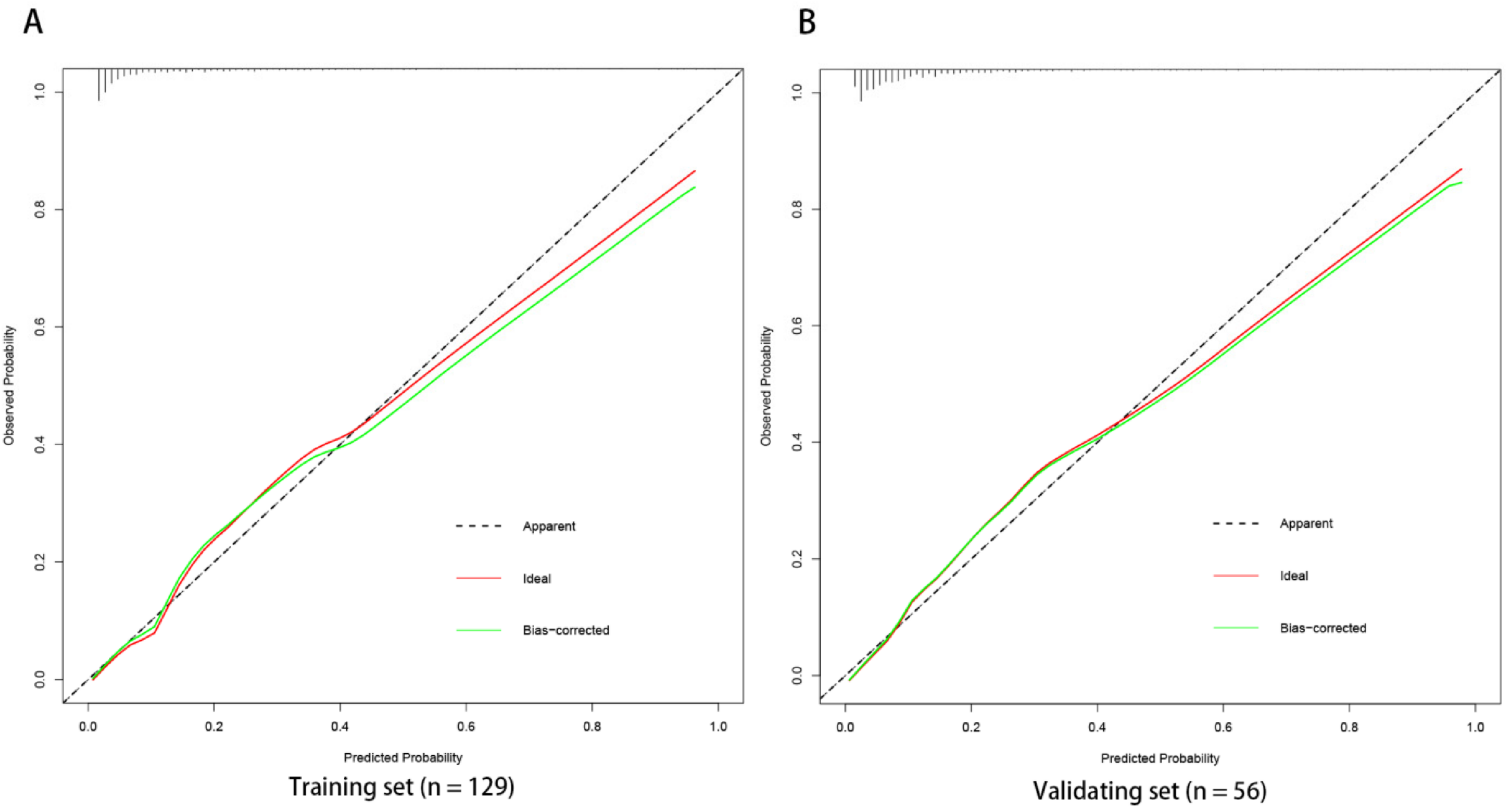
Calibration curve of nomogram prediction model the calibration curves of the training set **(A)** and verification set **(B)** of the nomogram model in this study are close to the ideal curves, suggesting that the model has good prediction efficiency.

**Figure 4:**
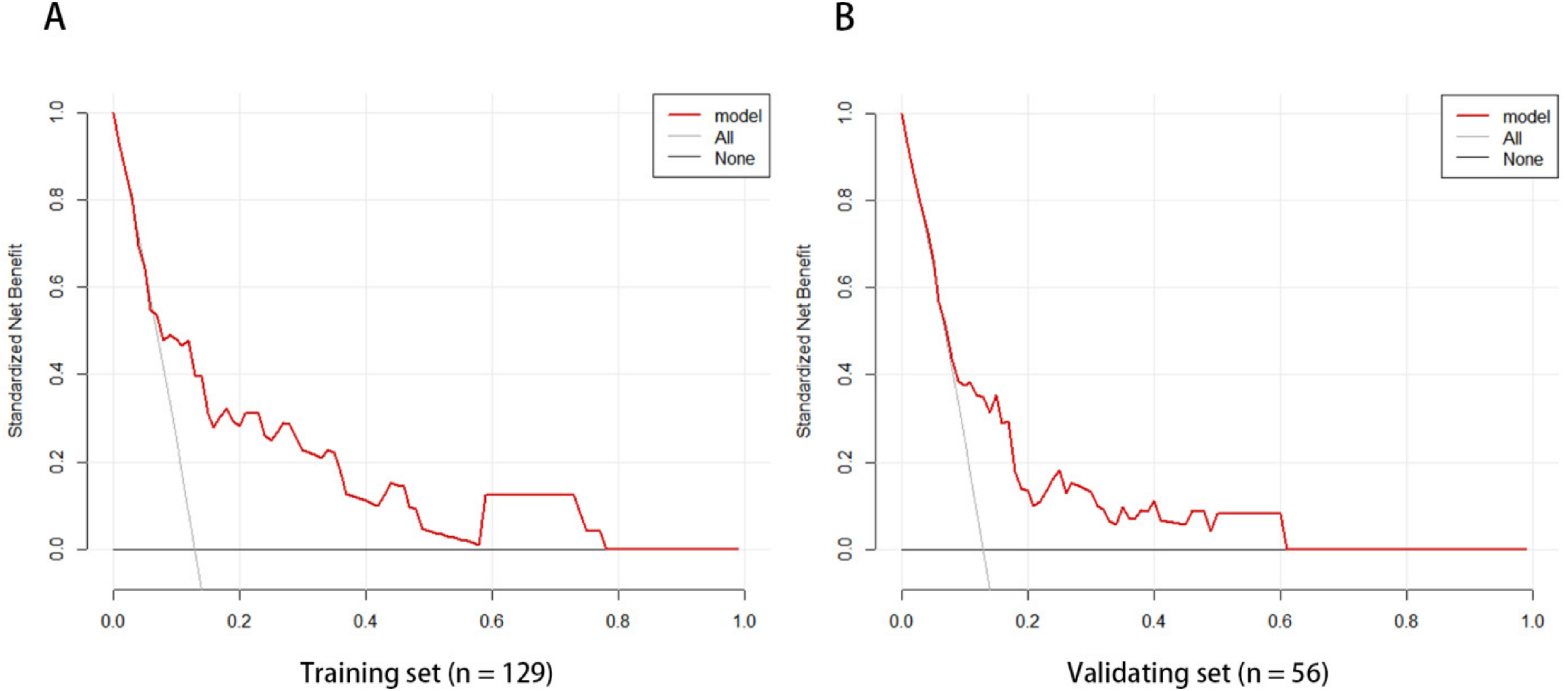
The model curves of the training set **(A)** and validation set **(B)** are both higher than the other two curves, which indicates that the prediction model has high clinical application value.

The ROC curve analysis of the line chart model showed that the AUC for predicting ROP reactivate in the training set was 0.810 (95% CI: 0.706–0.914), and in the validation set, the AUC was 0.756 (95% CI: 0.639–0.873) (Figure 2). The Hosmer-Leme show goodness-of-fit test indicated good model fit (*X*^2^ = 9.434, *P* = 0.31). Drawing the calibration curve of the line chart model showed that the calibration curves of the training set and validation set were close to the ideal curve, suggesting good predictive performance of the model (Figure 3). Decision curve analysis showed high clinical utility of the line chart model (Figure 4).

## Discussion

Retinopathy of prematurity (ROP) is the leading cause of preventable childhood blindness globally (15). It is characterized by the arrest of vascularization in the retina leading to a proliferative vitreoretinopathy (16).Globally, in 2010, an estimated 184,700 babies of 14.9 million preterm babies developed any stage of ROP, 20,000 of whom became blind (visual acuity <20/400) or severely visually impaired (visual acuity from <20/200 to ≥20/400) from ROP, and of whom 12,300 others developed mildmoderate visual impairment (visual acuity from <20/40 to ≥20/200) (17). The two main treatment options for ROP are laser and intravitreal injections of anti-VEG. Laser photocoagulation has been used as the standard treatment for ROP for decades, but it comes with significant side effects, including permanent retinal damage, visual field loss, retinal detachment, and refractive errors (18, 19). Subsequent studies have shown that compared to laser photocoagulation, intravitreal injection of anti-VEGF has higher efficacy and lower side effects (20–22). However, anti-VEGF therapy also carries the risk of ROP reactivate. In the report by Martínez-Castellanos et al., the reactivate rate of retinopathy of prematurity treated with bevacizumab was 6.8% (23). According to Süren et al., the reactivate rates of ROP treated with bevacizumab, ranibizumab, and aflibercept were 25.9%, 37.6%, and 23.2%, respectively (24). In the study by Valikodath et al., the reactivate rates of ROP treated with bevacizumab, ranibizumab, aflibercept, and concept were 4%–14%, 4.3%–52%, 7.7%, and 0–16.7%, respectively (7). In this study, the reactivate rate of ROP treated solely with anti-VEGF therapy was 13.0%, the ROP reactivation rates of ranibizumab, aflibercept and conbercept were 10.7%, 10.8% and 20%, which were basically consistent with previous literature reports.

ROP reactivate can lead to serious consequences such as retinal detachment, visual impairment, and permanent blindness. Therefore, early prediction of the risk of ROP reactivate can help clinicians formulate personalized follow-up and management strategies to improve the prognosis of affected children. Gupta et al. developed a quantitative scale for assessing the severity of retinopathy of prematurity regression using imaging and informatics data from nine tertiary referral centers in North America (25). They found that eyes requiring retreatment had higher ROP vascular severity scores at initial treatment compared to eyes receiving a single treatment. Compared to the quantitative scale mentioned above, the line chart visualizes complex statistical models, providing patients with a precise digital risk probability. This study is the first to develop a simple and user-friendly line chart model to predict ROP reactivate in the Asian population. Using single-factor and multi-factor logistic regression analysis, three potential predictors were identified. The line chart model includes three independent and measurable predictors: the number of red blood cell transfusions, use of PS 2 or more times, and preoperative fundus hemorrhage. The AUC of the ROC curves for the training set and validation set were 0.810 (95% CI: 0.706–0.914) and 0.756 (95% CI: 0.639–0.873), respectively, indicating good discrimination of the model. The Hosmer-Leme goodness-of-fit test and calibration curve demonstrate good consistency and accuracy between predicted and actual probabilities, while decision curve analysis shows the high clinical utility of the model.

Franz et al. randomly assigned 1,013 very low birth weight infants to a liberal red blood cell transfusion threshold group (*n* = 492) and a restrictive threshold group (*n* = 521) (26). They found that the incidence of severe retinopathy of prematurity requiring surgical intervention was 8.7% in the liberal threshold group and 7.7% in the restrictive threshold group. Glaser et al. conducted a retrospective cohort study of 12,565 preterm infants born at 22 + 0–28 + 6 weeks’ gestation (27). The study found a significantly higher frequency of red blood cell transfusion history in infants with ROP. After adjusting for confounding factors, red blood cell transfusion was positively associated with the incidence of ROP (OR 1.4, *P* < 0.001), progression of ROP (OR 2.1, *P* < 0.01), and ROP requiring treatment (OR 3.6, *P* < 0.001). Adult hemoglobin replacement for fetal hemoglobin may be a causal mechanism for ROP development, resulting in changes in oxygen affinity and increased oxygen release. Therefore, increased adult hemoglobin in transfused preterm infants may expose immature retinas to higher concentrations of oxygen and reactive oxygen species (28, 29). Multiple red blood cell transfusions may further cause retinal damage by promoting the accumulation of free iron and subsequent production of hydroxyl radicals (28, 30). Finally, pro-inflammatory and anti-inflammatory mediators, such as transfusion-related hemolysis, may promote retinopathy (31). The smaller the gestational age of premature infants, the more impaired their antioxidant protection and increased susceptibility. Therefore, the incidence of ROP appears to increase with decreasing gestational age (28).

Pulmonary surfactant plays a crucial role in the management of preterm infants. However, there is a close association between the incidence of ROP and multiple doses of pulmonary surfactant. Coshal et al. conducted a retrospective study of 8,024 preterm infants born at less than 28 weeks gestation (32). They found that infants receiving multiple doses of surfactant had lower gestational ages and weights compared to those receiving one or no doses of surfactant. Furthermore, the rates of severe neurological injury, bronchopulmonary dysplasia, and stage 3 or higher ROP were positively correlated with the dose of pulmonary surfactant. This finding suggests that receiving multiple doses of surfactant may be an effective marker of severe underlying respiratory immaturity. Wani et al. retrospectively analyzed the results of retinopathy of prematurity screening in 599 preterm infants. The study found that low birth weight (OR 22.86, 95% CI 3.86–134.82; *P* = 0.00), sepsis (OR 3.27, 95% CI 1.51–7.05; *P* = 0.002), and the need for surfactant were risk factors for severe ROP (33).

The immature retinal vascular system lacks smooth muscle, collagen, pericytes, and elastic fibers, immature retinal blood vessels may be fragile and more prone to rupture. Daniel et al. evaluated acute retinopathy of prematurity remotely. The study found a direct correlation between the presence of intraocular hemorrhage and the presence and severity of ROP, negatively correlated with gestational age and birth weight (34). Tong et al. found a significant association between retinal hemorrhage and the reactivate of aggressive retinopathy of prematurity (*P* = 0.01) (35).

There are currently many studies on risk factors for ROP reactivation, but the results vary widely. Patient factors related to immaturity include low gestational age, low birth weight, early PMA, and low Apgar scores (36). Disease factors include zone I ROP, extensive retinal neovascularization (13), preretinal hemorrhage before treatment (37), and aggressive posterior ROP (36). Due to the design limitations of a single-center retrospective study, although the three risk factors found in this study had the same findings in previous studies, there are still some risk factors that were not found to be statistically significant in this study. We will conduct further analysis and research in future large-sample multi-center studies.

In summary, we have developed a nomogram and web-based calculator demonstrating the good accuracy of predicting the risk of ROP recurrence after anti-VEGF treatment in our hospital population. Validation of this model in other populations and the development of similar models may allow early prediction of ROP recurrence, which will assist clinicians in formulating personalized follow-up and management strategies, thereby improving the prognosis of affected children. This study was a single-center retrospective study, with a small total sample size, possible selection bias, and a lack of external data to validate the established model. Therefore, in the future, it is necessary to conduct large-sample and multi-center studies to externally validate the model and further optimize the prediction model.

## Data Availability

The original contributions presented in the study are included in the article/Supplementary Material, further inquiries can be directed to the corresponding author.
