# Supplementary material for: Construction of a nomogram model to predict the risk of retinopathy of prematurity reactivate after intravitreal anti-vascular endothelial growth factor therapy: a retrospective study
Source: Front Pediatr. 2025 Jan 7;12:1440437. doi: 10.3389/fped.2024.1440437 (PMC11747279; doi:10.3389/fped.2024.1440437)
Supplement: Supplementary file 1 [file Table1.docx]

Supplementary Material

# Supplementary Figures and Tables

**Table 1** Clinical features in patients.

|  | Training set (n = 129) | | | | Validating set (n = 56) | | | |
| --- | --- | --- | --- | --- | --- | --- | --- | --- |
|  | No ROP recurrence group  **(n = 111)** | ROP recurrence group  **(n = 18)** | t/χ² | P-value | No ROP recurrence group **(n = 50)** | ROP recurrence group **(n = 6)** | t/χ² | P-value |
| GA(week, IQR) | 28.72 ± 1.86 | 27.62 ± 2.51 | t=2.223 | 0.028 | 28.73 ± 2.01 | 28.21 ± 1.67 | t=0.602 | 0.550 |
| BW (g, IQR) | 1067.34 ± 274.80 | 928.89 ± 248.31 | t=2.008 | 0.047 | 1097.60 ± 273.05 | 1105.00 ± 323.65 | t=-0.062 | 0.951 |
| Oxygen inhalation duration(day, IQR) | 35.74 ± 25.29 | 53.06 ± 36.85 | t=-1.922 | 0.069 | 33.33 ± 22.15 | 41.67 ± 33.40 | t=-0.824 | 0.414 |
| Invasive ventilator duration(day, IQR) | 11.01 ± 14.37 | 20.28 ± 23.04 | t=-2.307 | 0.023 | 7.87 ± 11.84 | 8.50 ± 10.69 | t=-0.124 | 0.902 |
| Non invasive ventilator duration(day, IQR) | 22.93 ± 12.20 | 26.56 ± 17.75 | t=-1.091 | 0.277 | 20.18 ± 19.26 | 24.67 ± 9.87 | t=-0.559 | 0.579 |
| Erythrocyte transfusion times(time, IQR) | 5.93 ± 4.03 | 9.89 ± 6.98 | t=-2.346 | 0.030 | 5.22 ± 3.74 | 6.00 ± 4.86 | t=-0.468 | 0.642 |
| Erythrocyte transfusion volume(ml, IQR) | 163.35 ± 112.45 | 263.00 ± 218.99 | t=-1.891 | 0.074 | 143.14 ± 101.87 | 162.17 ± 116.00 | t=-0.426 | 0.671 |
| HGB(g/L, IQR) | 106.14 ± 17.50 | 112.44 ± 17.31 | t=-1.419 | 0.158 | 102.73 ± 17.46 | 104.75 ± 9.55 | t=-0.276 | 0.783 |
| Male,n (%) | 72 (64.86) | 9 (50.00) | χ²=1.465 | 0.226 | 31 (62.00) | 2 (33.33) | χ²=0.827 | 0.363 |
| Natural conception,n(%) | 84 (75.68) | 12 (66.67) | χ²=0.272 | 0.602 | 45 (90.00) | 6 (100.00) | - | 1.000 |
| natural labour,n (%) | 43 (38.74) | 7 (38.89) | χ²=0.000 | 0.990 | 15 (30.00) | 3 (50.00) | χ²=0.279 | 0.597 |
| Multiple pregnancy ,n(%) | 27 (24.32) | 3 (16.67) | χ²=0.170 | 0.680 | 13 (26.00) | 1 (16.67) | χ²=0.000 | 1.000 |
| Gestational hypertension,n(%) | 46 (41.44) | 6 (33.33) | χ²=0.423 | 0.515 | 19 (38.00) | 1 (16.67) | χ²=0.336 | 0.562 |
| GDM, n (%) | 17 (15.32) | 4 (22.22) | χ²=0.154 | 0.695 | 5 (10.00) | 1 (16.67) | - | 0.511 |
| Chorioamnionitis,n (%) | 2 (1.80) | 0 (0.00) | - | 1.000 | 3 (6.00) | 0 (0.00) | - | 1.000 |
| Asphyxia, n (%) | 58 (52.25) | 8 (44.44) | χ²=0.378 | 0.539 | 25 (50.00) | 1 (16.67) | χ²=1.241 | 0.265 |
| NEC, n (%) | 16 (14.41) | 3 (16.67) | χ²=0.000 | 1.000 | 8 (16.00) | 1 (16.67) | - | 1.000 |
| RDS, n (%) | 110 (99.10) | 17 (94.44) | - | 0.261 | 48 (96.00) | 6 (100.00) | - | 1.000 |
| BPD, n (%) | 78 (70.27) | 16 (88.89) | χ²=1.856 | 0.173 | 28 (56.00) | 4 (66.67) | χ²=0.004 | 0.950 |
| Sepsis, n (%) | 92 (82.88) | 15 (83.33) | χ²=0.000 | 1.000 | 38 (76.00) | 4 (66.67) | χ²=0.000 | 1.000 |
| Pneumonia, n (%) | 32 (28.83) | 7 (38.89) | χ²=0.743 | 0.389 | 13 (26.00) | 1 (16.67) | χ²=0.000 | 1.000 |
| Intracranial hemorrhage, n (%) | 87 (78.38) | 16 (88.89) | χ²=0.510 | 0.475 | 42 (84.00) | 6 (100.00) | - | 0.578 |
| PDA, n (%) | 44 (39.64) | 8 (44.44) | χ²=0.149 | 0.700 | 20 (40.00) | 3 (50.00) | χ²=0.001 | 0.975 |
| ≥2 PS, n (%) | 27 (24.32) | 9 (50.00) | χ²=5.075 | 0.024 | 11 (22.00) | 2 (33.33) | χ²=0.012 | 0.913 |
| Zone preoperative screening , n (%) |  |  | χ²=3.014 | 0.083 |  |  | χ²=0.565 | 0.452 |
| Ⅰ | 38 (34.23) | 10 (55.56) |  |  | 13 (26.00) | 3 (50.00) |  |  |
| Ⅱ | 73 (65.77) | 8 (44.44) |  |  | 37 (74.00) | 3 (50.00) |  |  |
| Stage preoperative screening , n (%) |  |  | - | 0.079 |  |  | - | 0.253 |
| 1 | 5 (4.50) | 1 (5.56) |  |  | 4 (8.00) | 0 (0.00) |  |  |
| 2 | 47 (42.34) | 5 (27.78) |  |  | 24 (48.00) | 3 (50.00) |  |  |
| 3 | 58 (52.25) | 10 (55.56) |  |  | 22 (44.00) | 2 (33.33) |  |  |
| 4 | 1 (0.90) | 2 (11.11) |  |  | 0 (0.00) | 1 (16.67) |  |  |
| Plus preoperative screening , n (%) | 82 (73.87) | 16 (88.89) | - | 0.420 | 36(72.00) | 4 (66.67) | - | 0.191 |
| Bleed preoperative screening , n (%) | 8 (7.21) | 5 (27.78) | - | 0.019 | 4 (8.00) | 2 (33.33) | - | 0.119 |
| Surgical medication, n (%) |  |  | χ²=2.208 | 0.332 |  |  | - | 0.321 |
| Ranibizumab | 43 (38.74) | 7 (38.89) |  |  | 24 (48.00) | 1 (16.67) |  |  |
| Aflibercept | 41 (36.94) | 4 (22.22) |  |  | 17 (34.00) | 3 (50.00) |  |  |
| Conbercept | 27 (24.32) | 7 (38.89) |  |  | 9(18.00) | 2 (33.33) |  |  |

ROP,retinopathy of prematurity;GA, gestational age; BW, birth weight; GDM, gestation diabetes mellitus;NEC,necrotizing enterocolitis;RDS, neonatal respiratory distress syndrome;BPD, bronchopulmonary dysplasia; PDA, patent ductus arteriosus;PS, pulmonary surfactant.

**Table 2** Univariate and multivariate analysis of factors related to ROP recurrence.

|  | Univariable models | | Multivariable models | |
| --- | --- | --- | --- | --- |
|  | OR (95%CI) | P-value | OR (95%CI) | P-value |
| GA(week) | 0.70 (0.51 - 0.97) | 0.030 | 1.48 (0.86 - 2.56) | 0.155 |
| BW(g) | 0.99 (0.99 - 0.99) | 0.047 | 1.00 (0.99 - 1.00) | 0.140 |
| Invasive ventilator duration(day) | 1.03 (1.01 - 1.06) | 0.035 | 1.01 (0.97 - 1.05) | 0.098 |
| Erythrocyte transfusion times(time) | 1.16 (1.05 - 1.29) | 0.003 | 1.30 (1.05 - 1.61) | 0.018 |
| ≥2 PS(n ) | 3.11 (1.12 - 8.63) | 0.029 | 4.19 (1.07 - 16.47) | 0.040 |
| Bleed preoperative screening (n ) | 5.66 (1.57 - 20.45) | 0.008 | 14.09 (1.97 - 100.69) | 0.008 |

GA, gestational age; BW, birth weight;PS, pulmonary surfactant.


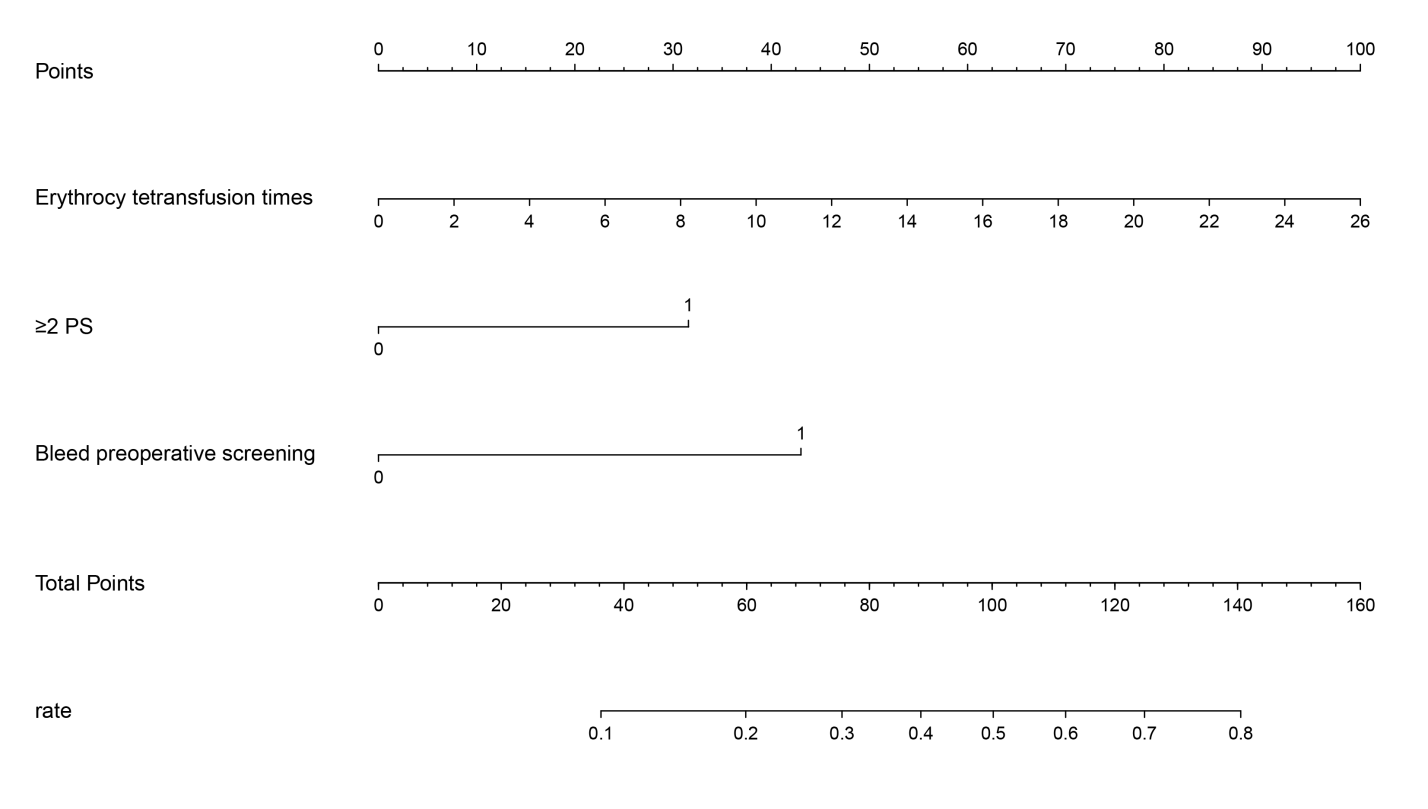


**Figure 1** Nomogram for ROP recurrence risk According to the variables, the corresponding scores are obtained, and the risk axis corresponding to the total score is obtained by adding the scores of each variable, so that the risk of ROP recurrence can be obtained.


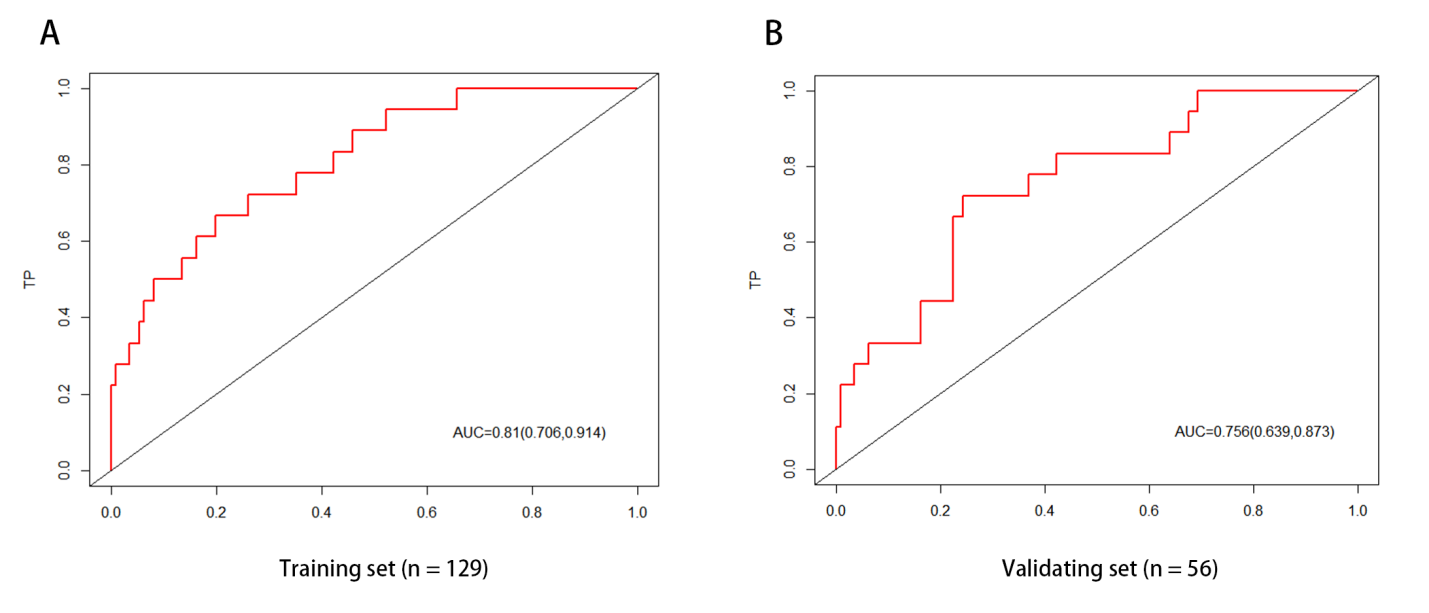


**Figure 2** ROC curve of nomogram prediction model The AUCs of the nomogram model in the training set and the verification set in predicting ROP recurrence is 0.810 and 0.756, respectively, which indicates that the model has good discrimination ability.


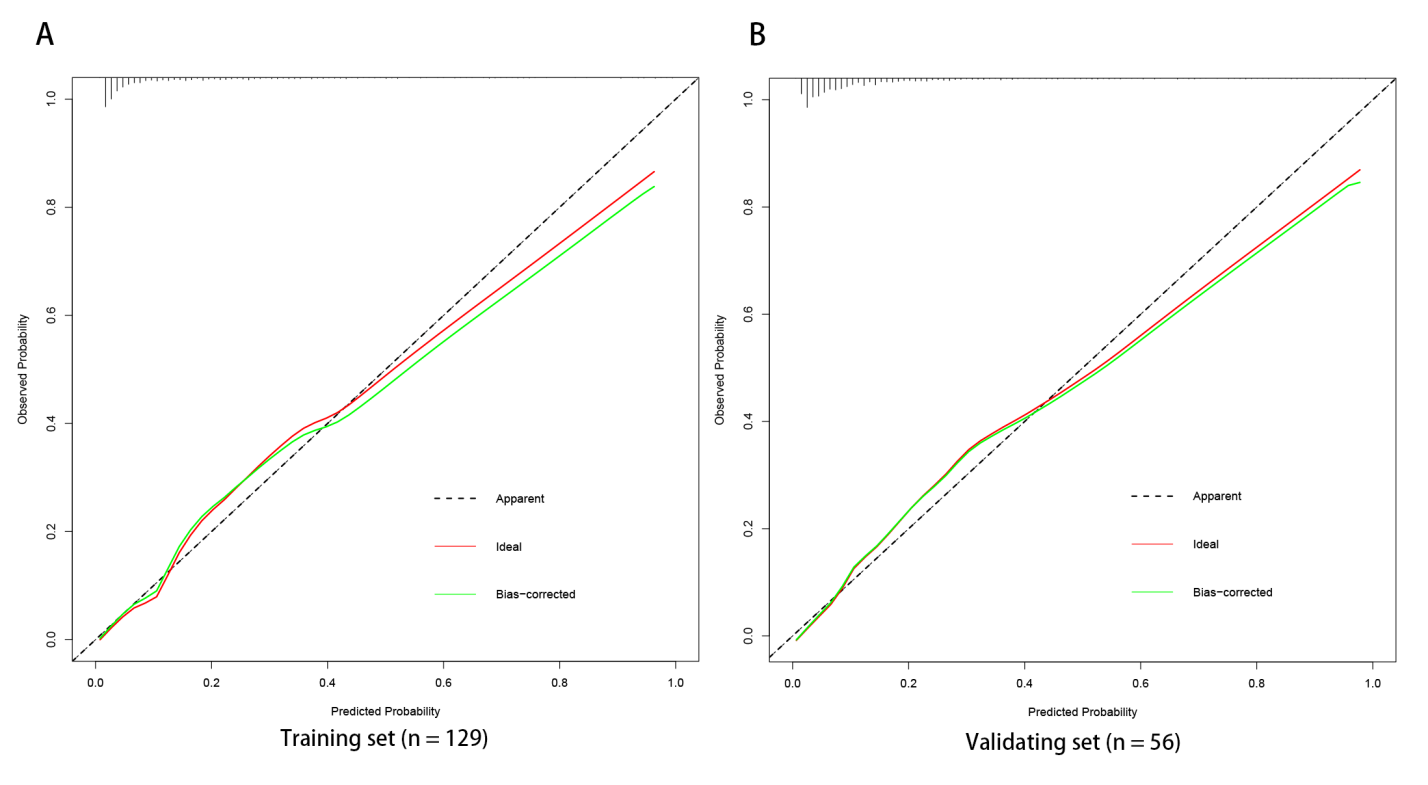


**Figure 3** Calibration curve of nomogram prediction model The calibration curves of the training set and verification set of the nomogram model in this study are close to the ideal curves, suggesting that the model has good prediction efficiency.


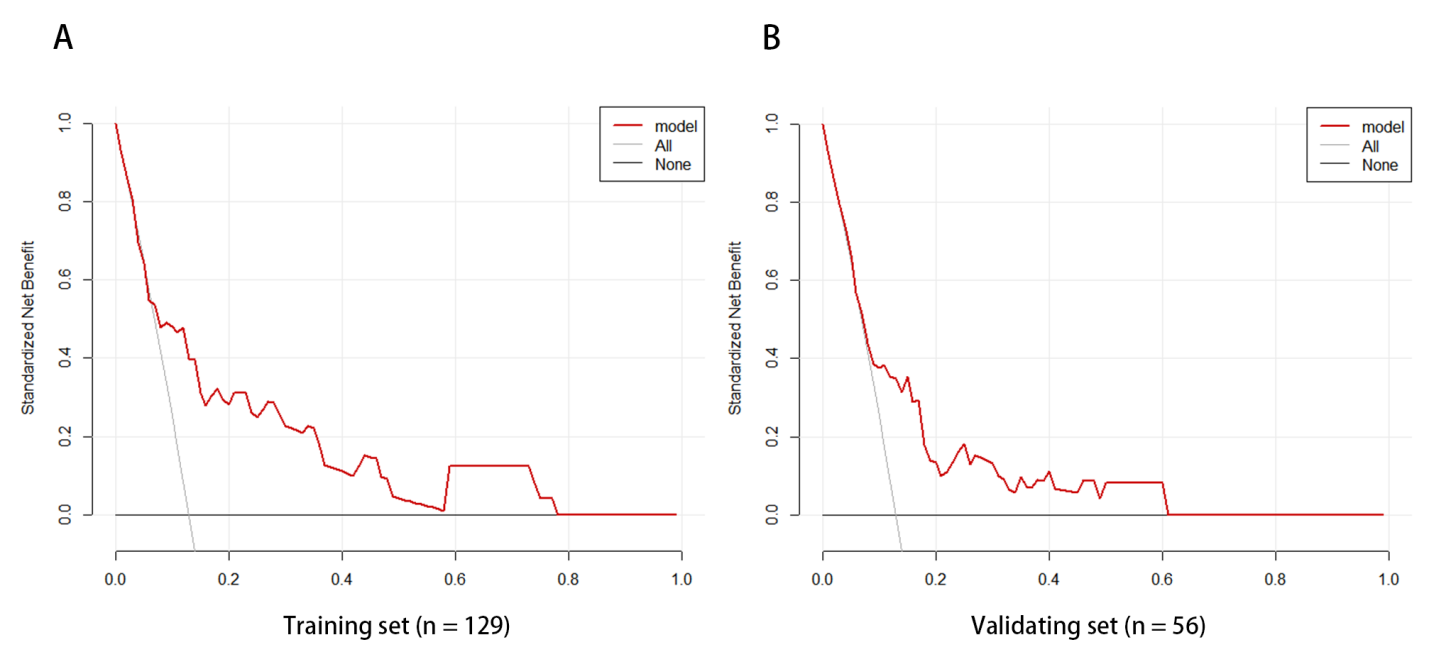


**Figure 4** Decision curve analysis of nomogram prediction model The model curve is higher than the other two curves, which indicates that the predictive model has high clinical application value.
